# The new Japanese regulation on human/non-human chimeras: should we worry?

**DOI:** 10.5935/1518-0557.20200045

**Published:** 2021

**Authors:** Vera Lúcia Raposo

**Affiliations:** 1 Faculty of Law of Macao University, Macao, China; 2 Faculty of Law of Coimbra University, Coimbra, Portugal

**Keywords:** chimeras, human stem cells, brain cells, human animals, non-human animals, fundamental rights

## Abstract

In March 2019 Japan modified its norms regarding research with human/non-human chimeras. The amended rules allow the creation of chimeras with human brain cells, and the subsequent transfer of the resulting creature to an uterus, where it can develop for more than 14 days, eventually until term. At this moment, the real consequences of this new regulation in actual research are still uncertain. However, many concerning issues have already been identified. This paper will start by addressing traditional topics involving this practice: the use of non-human animals in research, the use of human stem cells in scientific experimentation and the creation of human/non-human chimeras. Subsequently, it will analyze the new concerning issues brought on by the 2019 amendment: the use of human brain cells, the transfer of the chimera to an uterus and its development for more than 14 days, and the possibility of using animals which present close similarities with humans. In the end, the paper will conclude that in spite of the legal and ethical hazards that this new regulation might carry, it should be allowed under strict scrutiny.

## 1. THE RECENT LEGAL CHANGE IN JAPAN

Since some years ago, Japan allows research involving the creation of human/non-human (HNH) chimeric embryos introducing human cells in non-human (NH) animals,subject to certain conditions (Mizuno *et al*., 2015). According to the 2001 Guidelines for Handling of a Specified Embryo (Ministry of Education, Culture, Sports, Science and Technology, 2001), human-animal chimeric embryos can only be created for basic research regarding the creation of organs to be transplanted into humans; the referred embryos are not allowed to develop after the 14^th^ day, that is, when the primitive streak comes around; and they cannot be transferred into a human or animal uterus.

However, this issue has recently got more complex. The most recent Japanese regulation, issued on March 1, 2019 (Ministry of Education, Culture, Sports, Science and Technology, 2019), came to largely flexibilize this research in order to facilitate the creation of human organs inside HNH chimeras (Cyranoski, 2019; Sawai *et al*., 2019; Zimmer, 2019). In order to accomplish that aim, practices that used to be banned in Japan are now allowed.

One of the most significant changes is the newly-allowed creation of chimeras with human brain cells, in contrast with the previous regulation that expressly banned the use of these particular cells. The prior prohibition was based on the belief that “producing a brain derived from human cells in an animal body may have an effect on animal behavior, and should be regulated even at stages before individuals are generated” (Mizuno *et al*., 2015). The new regulation is based in a study carried out by the Japan’s Ministry of Education, Culture, Sports, Science and Technology, that concluded to be very unlikely that animals provided with some brain cells could acquire human brain functions (Zimmer, 2019). Based on these findings, the practice is now allowed.

Moreover, according with the current Japanese regulation, there is no fixed time limit for the development of the resulting entity; it depends on the study’s objective, the type of animal used, and the organ to be produced. All these elements must be assessed in order to establish for how long can the study last. This solution is substantially different from the previous one, which only allowed the resulting being to develop until the 14^th^ day.

Therefore, Japanese researchers are now authorized to create HNH chimeras with human brain cells, to transfer the product into an uterus and to let it develop for a period of time that can surpass 14 days, in order to obtain human organs. According to the representatives from the Japanese Ministry, this legal update does not raise special ethical or legal concerns because “there is technically zero risk of producing a new organism mixing human and animal elements under the research” (The Japan Times, 2019).

At this moment, the real consequences of this new regulation in actual researchis still uncertain. But, many concerning issues have already been raised.

## 2. TRADITIONAL ISSUES IN RESEARCH INVOLVING HNH CHIMERAS

### 2.1. The use of NH animals in scientific experimentation

According to the existing laws, animals (the concept of “animals”, as used in the text, refers to non-human animals) are not holders of rights, but are the object of rights, because animals are *res,* i.e., things (Bryant, 2008). This traditional perspective on the legal status of animals has changed in more recent times, due to the legal recognition that animals are sentient beings in some countries: in Austria see § 285 a of the Allgemeines Bürgerliches Gesetzbuch (ABGB, i.e., the General Civil Code), from 1812, as revised in 1988 (‘Animals are not things; they are protected by special laws. The provisions in force for the things apply to animals only if no contrary regulation exists’ - author’s translation); in Germany see Section 90 a) of the Bürgerliches Gesetzbuch (BGB, i.e., the Civil Code), as revised in 2002 (‘Animals are not things. They are protected by special statutes. They are governed by the provisions that apply to things, with the necessary modifications, except insofar as otherwise provided’ - author’s translation); in Switzerland see Art. 641a of the Schweizerisches Zivilgesetzbuch (ZGB, i.e., the Swiss Civil Code), as revised in 2002 (‘1- Animals are not objects. 2- Where no special provisions exist for animals, they are subject to the provisions governing objects’- author’s translation); in Portugal see Article 201.º-B of the Código Civil (Civil Code), as revised in 2017 (‘Animals are living beings endowed with sensibility and the object of legal protection by virtue of their nature’- author’s translation). From their qualification as sentient beings, a special legal protection is derived, not as strong as the one provided to human beings, but much stronger than the one assigned to things.

The new understanding on the legal status of animals imposed different legal solutions in several domains. For instance, the use of animals in scientific experimentation has been the object of increased restrictions over the years, based on the concern for their welfare (Deutsche Forschungsgemeinschaft, 2019). In the particular case of HNH chimeras, even if there is no suffering in the creation of chimeras, there is data demonstrating that some chimeric animals suffer from negative symptoms (Porsdam Mann *et al*., 2019), and get more prone to be infected by human virus, such as HIV (Berges & Rowan, 2011).

### 2.2. Research involving human embryonic stem cells (hESCs)

Research with human stem cells - either human embryonic stem cells (hESCs) or induced pluripotent stem cells (iPSCs) - raises several legal and ethical issues, for the very simple fact that those cells are human (King & Perrin, 2014).

In Europe, a crucial norm to assess research with hESCs is Article 17 of the Convention for the Protection of Human Rights and Dignity of the Human Being with regard to the Application of Biology and Medicine. According to this norm, it is forbidden to create *in vitro* human embryos for research purposes, including the extraction of hESCs (Giammarinaro, 1999; Raposo, 2012; Raposo, 2014; Romeo Casabona, 2008). A *contrario sensu*, only human embryos created for purposes other than experimentation, which subsequently became useless (surplus embryos), can be used in scientific research.

The question of informed consent in the use of human cells, embryonic or not, is a crucial one. The cells donors must be enlightened about the prospective uses of their cells. In the case under analysis, they must be informed about the several legal and ethical issues involved in the creation of HNH chimeras with their brain cells, a scenario which obviously raises concerns unknown in other types of research. When hESCs are used, consent must be provided by the embryo’s parents (Lo *et al*., 2003; 2004), but some have argued that the regular informed consent model should be adapted to embryo research (Casey & Adams, 2001). The main problem is to define who the parents are in this regard: the legal parents, whether or not genetic ones, or the gamete donors (in case they exist)? Usually gamete donors are prevented from making any decision in regards of embryos generated from their gametes, but this solution is not suitable when embryo experimentation is involved (Raposo, 2014); since in this case it is the donors’ DNA that will be involved in the creation of the chimera, and so the donors should also be required to consent. Lo *et al*. (2003; 2004) advocate for the consent of both the gamete donors and the legal parents (the people undergoing reproductive treatments). Moreover, the authors state that both donors - of oocytes and sperm - need to provide their consent for research involving the embryos created with their genetic material, but in the case of females, the requirements for consent should be more demanding because of the higher health risks involved in the extraction of oocytes.

### 2.3. The creation of HNH chimeras

#### *2.3.1.* Objections to the creation of HNH chimeras

The HNH chimera is an entity composed by human and animal cells (Hermerén, 2015), created by introducing human cells into an NH embryo. This paper uses the concept of ‘chimera’ in accordance with the definition provided by the 2011 report of the Academy of Medical Sciences (2011): an entity ‘formed by mixing together whole cells originating from different organisms. The new organism that results is made up of a ‘patchwork’ of cells from the two different sources. Each cell of a chimera contains genes from only one of the organisms from which it is made’. In contrast, hybrids are defined as ‘animals formed by the fertilization of an egg of one species by the sperm of a different species’ (Academy of Medical Sciences, 2011). The latter are the ‘true hybrids’. There are also ‘inter-specific cell hybrids’, which are ‘created by the fusion of cells from two different species (e.g. human cells fused with mouse cells)’ (Academy of Medical Sciences, 2011).

The creation of chimeras can envisage different purposes (Eberl & Ballard, 2009), such as the creation of organs to be developed into the chimera’s body and later on used in humans (Rashid *et al*., 2014), and the development of new drugs (Bhan *et al*., 2010); it can contribute to stem cell research (Levine & Grabel, 2017) and to study the progression of human diseases (De Los Angeles *et al*., 2018), among many other purposes.

The creation of HNH chimeras has for long been the object of criticism (Hinterberger, 2018; Hyun, 2016; Hübner, 2018), but most of the objections lack proper substance. It has been said that the mixture of species equates to playing God (The Danish Council of Ethics, 2007; Peters, 2007), that is, it would change the natural order of things. However, under this reasoning pretty much every aspect of our modern life (the use of medication, surgical interventions, travelling by plane) would have to be considered a modification of the natural order of things (we are not supposed to flight, we are not supposed to survive diseases) and thus, all that would have to be prohibited (Koplin & Savulescu, 2019).

An additional objection is that by mixing species we would be depriving the human being from that special value that for long has been granted to us (The Danish Council of Ethics, 2007), ultimately undermining human dignity (The Danish Council of Ethics, 2007), and the dignity of the non-human creature (Karpowicz *et al*., 2004). This issue produces relevant and complex questions, not only associated to the value of human beings but also to the value that should be granted to chimeras. The paper won’t discuss these questions. But it is still worth underlying that it is not enough to accuse the creation of HNH chimeras of endangering (human and non-human) dignity, as it also necessary to clearly identify in which exact way that happens.

Discussions involving the creation of such entities have also accused chimeras of blurring the line between the two species (Mizuno *et al*., 2015; Robert & Baylis, 2003; The Danish Council of Ethics, 2007), making it difficult to differentiate them and thus to justify the different legal and ethical status provided to human beings. However, from the opposite site it has been stated that ‘the interspecies boundary that exists between humans and livestock is sufficiently high that it is quite unlikely that acute chimerization of all aspects of the resulting animal will occur’ (Hyun, 2016). So, this argument is far from consensual.

#### *2.3.2.* Different regulation models

The creation of chimeras is forbidden in some jurisdictions, while allowed in others, under specific constraints.

One of the most restrictive regulations in this regard is the German one. The Embryo Protection Act (Embryonenschutzgesetz-ESchG) bans the creation of any kind of chimera, most likely due to the influence of the horrors associated with human experimentation committed during the Nazi past.

In Australia, the Prohibition of Human Cloning for Reproduction Act, from 2002, banns chimeras created by the introduction of animal cells in human embryos, but not the ones created via the introduction of human cells in animal embryos.

Likewise, in Canada, the Assisted Human Reproduction Act, from 2004, bans chimeras derived from the introduction of animal cells into human embryos, but not the opposite. However, many agencies dealing with research funding have adopted an extensive interpretation of the ban, in the end banning all types of chimeras (Koplin & Savulescu, 2019).

Federal US laws do not prohibit the creation of chimeras. Nonetheless, there are some restrictions on federal funding for research by the National Institute of Health (NIH). A moratorium was implemented in 2015, regarding the allocation of funds for research ‘in which human pluripotent cells are introduced into non-human vertebrate animal pre-gastrulation stage embryos’ (NIH, 2015). The moratoria only covered certain types of HNH chimeras (Hyun, 2016), and even in what regards the modalities under the moratoria, private funds could still be used. However, the measure was object of severe criticism (Sharma *et al*., 2015) and in 2016 it was substituted by a specific review for some types of research (NIH, 2016).

In the UK, the Human Fertilization and Embryology Act (HFEA) of 2008, in its current version allows the creation of the so called ‘human admix embryos’ (Hinterberger, 2016; Ogbogu *et al*., 2008). This concept refers to embryos created under the methods established in Subsection 4A (6) of the HFEA; under the condition that ‘the animal DNA is not predominant’. However, there is no legal definition of what ‘predominant’ is, so one can question whether is refers to any case in which human DNA surpasses non-human DNA or only to cases where the former largely surpasses the latter. Human admixed embryos whose genetic material is predominantly animal are not regulated by the HFEA and actually have no specific regulation in the UK.

## 3. PROBLEMATIC ISSUES RAISED BY THE NEW JAPANESE REGULATION

### 3.1. The specific type of human cells used

The 2019 amendment came to allow the use of human brain cells to generate HNH chimeras, eliminating the existing ban. This is not the first concerning incident in this regard. For instance, a study performed in the University of Rochester, and reported in a 2013 paper (Han *et al*., 2013), was able to create mice with human glial cells (that is, thinking cells) in their brains. Test results showed that the chimeric mice were much smarter than their peers. In the first half of 2019, a Chinese study involving HNH chimeras created with brain cells was made public (Shi *et al*., 2019; Shi & Su, 2019). The study involved the implantation of the human MCPH1 gene (a very relevant element for brain development, whose mutation can result in microencephaly) in eleven rhesus monkeys. The results showed that the brains of these chimeric monkeys presented some features similar to human brains in their development, for instance, better short-term memory and a longer time to develop, just like in humans.

The main peril is the creation of animals with human brain functions and the hypothetic consequences this feature may have on animal behaviour (Farahany *et al*., 2018; Sawai *et al*., 2017a; 2017b). In particular, the question is whether the introduction of human brain cells into the animal brain would grant them capacities considered exclusive human attributes. Accordingly, the British Academy of Medical Sciences concluded that the transplantation of human brain cells into animals, in such a way that they adopt a ‘“human-like” behaviour’ (Academy of Medical Sciences, 2011, p. 9), should not be authorized.

We still lack the answer for some basic scientific questions. For instance, how many human brain cells would be necessary for an animal to develop human behaviours and what kind of behaviours would those be? In the Rochester study quoted above, researchers argued that there was nothing particularly human about these mice with human glial cells in their brains: ‘This does not provide the animals with additional capabilities that could in any way be ascribed to or perceived as specifically human. Rather, the human cells are simply improving the efficiency of the mouse’s own neural networks. It’s still a mouse’. (Steve Goldman, quoted in Loike, 2015).

We also ignore whether there is a specific moment in time when human brain cells must be introduced into NH embryos in order to achieve particular cognitive skills. The moment in which the human cells are introduced in the NH being is crucial for the subsequent development of the chimera. If it happens postnatally the transferred cells will not substantially modify the animal structure. In contrast, if it happens during the foetal stage, and especially during the embryonic stage, the human cells will have the power to substantially shape the genomics of the animal (Hyun, 2016). But we still ignore if particular consequences are connected with specific lapses of time.

At this stage, we know that the introduction of human neural stem cells changes the brain of NH animals (Eberl & Ballard, 2009; Han *et al*., 2013; Shi *et al*., 2019; Shi & Su, 2019), but it is still unknown whether this is sufficient to make them develop the same skills as humans in what regards reasoning and self-consciousness (Cyranoski, 2019). Some years ago, the British Academy of Medical Sciences stated that ‘merely demonstrating quantitative enhancement of one aspect of an animal’s cognitive function does not imply its cognitive capacity is approaching that of the human’ (Academy of Medical Sciences, 2011, p. 47). Likewise, in a study, Hyun (2016) stated that no matter how developed a HNH chimera’s brain could become, it will never reach the level of self-consciousness characteristic of humans, ‘as it takes several years to develop in infant brains that are 100% human and only under the right social and nurturing conditions of child-rearing’. Hyun (2016) qualifies as ‘anthropocentric arrogance’ the belief that the mere existence of human brain cells into an NH brain could magically make that animal human or quasi-human. Even if higher cognitive capacities are not enough to require these enhanced chimeras to be treated as humans, they might still be able to change the moral status of these entities (Porsdam Mann *et al*., 2019), and thus also its legal status.

If the resulting chimera is considered human (because indeed it has human cells), or even if it is merely granted a higher status than the one provided for NH animals, the grounds, content and purposes of research involving these creatures will have to be reassessed. In particular, a proper ethical and legal base for its elimination at the end of the research will be required (Farahany *et al*., 2018). In the past, animals used in research were euthanized, but currently some of them (namely chimpanzees) are sent to sanctuaries to live the rest of their lives (Gagliardo-Silver, 2019).

Ultimately, it might involve the recognition of dignity and some sort of legal rights to the chimera.

### 3.2. The transfer of the resulting chimera into a uterus and its development after the 14^th^ day

The transfer of the resulting chimera into the uterus of an animal is a particularly problematic feature of the Japanese regulation.

Even the UK, that has one of the most flexible regulations in this regard (Home Office, 2016), does not allow the placement of a chimera (the so called ‘human admixed embryos’) into a uterus, neither animal, nor human (Subsections 4A(1) and 4A(4) HFEA, 2008). This prohibition seems to include the placing of human embryos into genetically modified animals in order to create a human uterus inside them (Academy of Medical Sciences, 2011).

The transfer of the chimeric being would not be so problematic if there were a temporal threshold. Until the last amendment, the Japanese regulation did not allow the chimeric embryo to develop for more than 14 days (Cavaliere, 2017), which corresponds to a rule commonly accepted worldwide. This 14-day rule derives from the Warnock Report, that forbids research on human embryos after the 14^th^ day of existence, under the argument that before 14 days we cannot consider it to be an ‘individual’, because the primitive streak is not yet formed and an embryonic division is still possible (Warnock, 2000) (the two events - the 14^th^ day and the primitive streak - happen almost simultaneously, and the 14^th^ day is usually indicated as a decisive temporal moment, precisely because of the primitive streak). Before that temporal threshold, the existing creature has no relevant moral or legal status, and it is not unlawful to use it in destructive scientific experiments. According to the British Academy of Medical Sciences (2011, p. 9), HNH chimeric embryos should not be allowed to develop ‘beyond 14 days of development or the first signs of primitive streak development (whichever occurs first); unless there is persuasive evidence that the fate of the implanted (human) cells will not lead to “sensitive” phenotypic changes in the developing foetus’.

The revised Japanese regulation admits the development of the chimera after the 14^th^ day limit (in order to allow organs to develop), and eventually it might be brought to term. However, we do not even know whether and how this chimeric embryo will develop.

The transfer of the resulting chimera to an animal uterus and its eventual birth should not be totally banned, but it can be allowed having in consideration the relevance of the aim, which, in turn, should be an aim of relevant importance, not achievable by other means.

### 3.3. The specific type of NH animal recipient

Within the discussion involving HNH chimeras a strong concern is the type of NH animal used to be the receptor of the human cells. To imagine a chimeric mouse does not cause huge concern, but to do the same with apes does. The mouse is too different (even though not that different as we might think) from a human person, whereas an ape is too similar (Capps, 2017).

‘If the recipient blastocyst were from an animal that is evolutionarily closer to a human, the potential for human contributions would appear to be greater (…) The need for blastocysts from larger mammals [than mice] would need to be very clearly justified, and nonhuman primate blastocysts should not be used at this time’ (National Research Council & Institute of Medicine, 2005).

When the use of human brain cells is involved, the main feature to have in consideration is the size of the animal’s brain. If experiments of this kind take place in animals with larger brains (as it is the case of great apes), it increases the chance of having those brains operating in ways similar to human ones (Academy of Medical Sciences, 2011), and thus the risks of creating a creature too close to humans becomes more stringent. The size of the animal’s brain was one of the main reasons to support the study proposed by Dr. Irving Weissman, from Stanford University (USA), involving the substitution of part of the brain cells of a mouse for human brain cells (Greely *et al*., 2007a; 2007b). Due to its small size, the working group that analyzed the study considered that it could never develop cognitive skills such as that of human brains. However, the fact that the small size of the receptor makes it difficult for the brain to grow as in a human being has advantages, but also has its drawbacks, because it is difficult to use the brain of the HNH chimera as a suitable model for humans in research (Farahany *et al*., 2018).

## 4. IS THIS RESEARCH WRONG?

The new Japanese regulation involves features that might create pressing concerns, maybe not when considered separately, but certainly when all of them are combined, because they might allow the birth of a creature with enhanced cognitive skills, eventually closer to humans. We must be prepared for a scenario in which a chimeric creature, with a partial human brain (and eventually some physiognomic features not that different from the humans, as it will happen if the receptor of the human cells is an ape or a chimpanzee) is allowed to be born in order to pursue post-birth studies.

Most people would be disgusted with the mere thought of mixing humans with animals. ‘A plausibly "thin" explanation for the intuitive "yuck" response [which in turn could be included in the wisdom of repugnance of Kass (1998)], is that the creation of ...creatures from human materials evokes the idea of bestiality -an act widely regarded as amoral abomination, because of its degrading character’ (Robert & Baylis, 2003).

From an ethical and legal standpoint, several issues must be addressed: can these HNH chimeras aspire to an autonomous legal status (Hübner, 2018)? Can they hold rights (Hübner, 2018)? Would do they have to provide consent for the kind of experiment that they are subjected to (Hübner, 2018)?

The answer to those questions implies the previous definition of the kind of change the introduction of human brain cells can operate on the NH embryo, namely to establish if such change is relevant enough to transform the NH embryo (the chimera) into a substantially different moral and legal entity (Eberl & Ballard, 2009). At this point, we do know that the introduction of human neural stem cells changes the brain of NH animals, but it is still unknown whether this is enough to make them develop the same skills as humans, in regards of reasoning and self-consciousness.

The issues at stake are abundant and complex, but the benefits of such study should not be forgotten. The use of human brain cells in living extra utero NH animal models may be an essential and irreplaceable scientific tool to understand human neurology, in order to combat diseases currently still untreatable, such as Alzheimer, Parkinson or dementia. An answer regarding the legal admissibility of these experiments will have to be achieved on a case-by-case basis (Farahany *et al*., 2018).
